# The VA Normative Aging Study: Reflecting Six Decades of Aging Research and Innovating Forward

**DOI:** 10.1093/geroni/igaf122.1456

**Published:** 2025-12-31

**Authors:** Lewina Lee, Carolyn Aldwin, David Almeida

## Abstract

Deeply-phenotyped longitudinal cohort studies have been the cornerstone of aging research. They collect rich data on biopsychosocial processes, including biomarker and neurocognitive assessments, and embed time-intensive experience sampling protocols that enable rigorous designs to inform etiological knowledge and developmental processes underlying health and well-being outcomes. The Veterans Affairs Normative Aging Study (NAS), initiated to distinguish between illness and normal aging, is among first longitudinal aging studies initiated in the US in the mid-20th century and has evolved into a deeply-phenotyped study over 60+ years. This symposium will consider the scientific contributions of NAS to the fields of stress, personality, and aging. It will also examine recent innovations aimed to augment the scientific value of aging cohorts nearing the end of participants’ lifespan. Dr. Spiro will describe the evolution of NAS from its inception in 1963 to become an exemplar deeply-phenotyped study through substantial efforts to cultivate a robust psychosocial research program. Dr. Aldwin will synthesize findings and lessons learned from four decades of stress and coping research using NAS. Turning to methodological developments, Dr. Mroczek will illustrate how a multi-study coordinated analysis framework can leverage the strengths of aging cohort studies to enhance scientific replicability. Dr. Marino will demonstrate the use of administrative data linkages to enrich NAS with historical data on childhood environmental (lead) exposure and adding siblings into the cohort. Dr. Almeida, an expert on studying stress and aging using intensive longitudinal designs, will discuss the collective wisdom gleaned and help envision the next generation of research. Aging Veterans: Effects of Military Service across the Life Course Interest Group Sponsored Symposium

